# Association of identification of facility and transportation for childbirth with institutional delivery in high priority districts of Uttar Pradesh, India

**DOI:** 10.1186/s12884-021-04187-5

**Published:** 2021-10-27

**Authors:** Divya Rajvanshi, John Anthony, Vasanthakumar Namasivayam, Bidyadhar Dehury, Ramesh Banadakoppa Manjappa, Ravi Prakash, Dhanunjaya Rao Chintada, Shagun Khare, Lisa Avery, Maryanne Crockett, Shajy Isac, Marissa Becker, James Blanchard, Shiva Halli

**Affiliations:** 1 India Health Action Trust, Lucknow, Uttar Pradesh/ New Delhi India; 2 Institute of Global Public Health, University of Manitoba, Winnipeg, Canada

**Keywords:** Institutional delivery, Birth preparedness, Antenatal care, Uttar Pradesh, Frontline worker

## Abstract

**Background:**

Timely and skilled care is key to reducing maternal and neonatal mortality. Birth preparedness involves preparation for safe childbirth during the antenatal period to reach the appropriate health facility for ensuring safe delivery. Hence, understanding the factors associated with birth preparedness and its significance for safe delivery is essential. This paper aims to assess the levels of birth preparedness, its determinants and association with institutional deliveries in High Priority Districts of Uttar Pradesh, India.

**Methods:**

A community-based cross-sectional survey was conducted between June–October 2018 in the rural areas of 25 high priority districts of Uttar Pradesh, India. Simple random sampling was used to select 40 blocks among 294 blocks in 25 districts and 2646 primary sampling units within the selected blocks. The survey interviewed 9458 women who had a delivery 2 months prior to the survey. Descriptive statistics were included to characterize the study population. Multivariable logistic regression analyses were performed to identify the determinants of birth preparedness and to examine the association of birth preparedness with institutional delivery.

**Results:**

Among the 9458 respondents, 61.8% had birth preparedness (both facility and transportation identified) and 79.1% delivered in a health facility. Women in other caste category (aOR = 1.24, CI 1.06–1.45) and those with 10 or more years of education (aOR = 1.68, CI 1.46–1.92) were more likely to have birth preparedness. Antenatal care (ANC) service uptake related factors like early registration for ANC (aOR = 1.14, CI 1.04–1.25) and three or more front line worker contacts (aOR = 1.61, CI 1.46–1.79) were also found to be significantly associated with birth preparedness. The adjusted multivariate model showed that those who identified both facility and transport were seven times more likely to undergo delivery in a health facility (aOR = 7.00, CI 6.07–8.08).

**Conclusion:**

The results indicate the need for focussing on marginalized groups for improving birth preparedness. Increasing ANC registration in the first trimester of pregnancy, improving frontline worker contact, and optimum utilization of antenatal care check-ups for effective counselling on birth preparedness along with system level improvements could improve birth preparedness and consequently institutional delivery rates in Uttar Pradesh, India.

**Supplementary Information:**

The online version contains supplementary material available at 10.1186/s12884-021-04187-5.

## Introduction

The World Health Organization (WHO) estimates that around 295,000 maternal deaths occurred globally in 2017 and 12% of these deaths occurred in India [1]. As per the latest available estimates in India, the Maternal Mortality Ratio (MMR) stood at 113 per 100,000 live births in 2016–18 [2] while the Neonatal Mortality Rate (NMR) was estimated to be 23 per 1000 live births in 2018 [3]. With an estimated population of almost 1.36 billion [4], India is a diverse country with significant inter-state and intra-state variations wherein the highest MMR has been recorded in the state of Assam (215 per 100,000 live births) and the lowest in Kerala state (43 per 100,000 live births). The NMR also varies substantially across the country with the state of Madhya Pradesh (35 per 1000 live births) having the highest NMR and Kerala state recording the lowest NMR (5 per 1000 live births). Uttar Pradesh is the most populous state in India with an estimated population of 235 million spread across 75 districts. It has the second highest MMR (197 per 100,000 live births) and NMR (32 per 1000 live births) in the country [1, 2]. In 2017, a national report on neo-natal mortality trends in India showed substantial inter-district variations within Uttar Pradesh ranging between 46.2 in Budaun district and 22.8 neonatal deaths per 1000 live births in Deoria district [5]. The alarming estimates call for additional targeted efforts to achieve the Sustainable Development Goals (SDG) of an MMR of 70 per 100,000 live births and an NMR of 12 per 1000 live births by 2030 in Uttar Pradesh.

Most of the maternal deaths occurring during pregnancy and childbirth are caused by excessive blood loss, high blood pressure followed by other direct causes like sepsis, unsafe abortion as well as indirect causes that are often preventable and highly manageable [1]. These deaths can be prevented by addressing the delays in deciding to seek care, reaching the appropriate health care institutions and receiving adequate and emergency care in these institutions [6, 7]. To address these delays and promote the timely use of skilled maternal and neonatal care, WHO and other international agencies recommended the strategy of Birth Preparedness and Complication Readiness (BPCR) which involves preparation for childbirth during pregnancy. A systematic review of randomized trials of BPCR interventions showed such interventions are effective in reducing the risk of maternal and neonatal mortality in low resource settings [8]. The definition of birth preparedness varies across different periods, sources and places, and may include a wide range of aspects which include identifying the location of the facility for birth, ensuring funds for birth-related expenses, identifying the mode of transport for reaching the identified facility, identification of blood donors, identification of birth companions, improving knowledge of danger signs among pregnant women and in some cases information of danger signs in newborns, preparing for skilled early postpartum care, obtaining HIV test during pregnancy as well as supply side interventions like well-equipped facilities and providers [9, 10]. While complication readiness is essential to ensure access to emergency obstetric care, the state of Uttar Pradesh in India is still fraught with high proportions of home births [11]. Hence, this study focusses on two core elements of birth preparedness (anytime during the pregnancy but before the onset of labour) i.e. identification of facility and transport for delivery which would solve for two delays of decision to seek care and reaching the appropriate health care facility.

Evidence from Sub-Saharan Africa suggests that education, parity, antenatal care visits, age, place of residence and gender are predictors of knowledge on the components of birth preparedness that include identifying a birth attendant, blood donor, place of child birth, saving money and knowledge of danger signs [12]. A study in Southern Ethiopia established that pregnant women who were pregnant for the first time and those availing antenatal care services were more likely to follow at least two of the five components of birth preparedness considered for the study i.e. identification of a skilled birth attendant, health facility, blood donor, transportation and saving money for child birth [13]. In Burkina Faso, a positive impact on the rate of institutional births was observed in districts with low institutional birth rate by providing birth preparedness related messages during antenatal visits [14]. Similarly, the odds of a favourable birth outcome increased three fold with adequate birth preparedness in Kenya [15].

Birth preparedness programs are being implemented in the state of Uttar Pradesh, India as part of the maternal health guidelines of the National Health Mission. A study in Uttar Pradesh evaluating the effectiveness of birth preparedness amongst pregnant women found that women who followed at least one of the birth preparedness steps which included identifying a health facility, an accompanying person with mother, arranging a transport and saving money, were 45% more likely to undergo institutional deliveries [16]. In another study in Uttar Pradesh, it was found that women who registered for Antenatal Care (ANC) in a hospital or with a doctor and whose husbands were involved in deciding the place of delivery were more likely to have followed at least 5 steps of birth preparedness such as thinking about measures to be taken in case of life danger, being aware of the delivery date, saving money, discussing plans with close family members, keeping ready clean cotton and other necessities, identifying a hospital, arranging transportation and arranging doctor or *Dai* (a traditional birth attendant) [17]. Lastly, another study conducted in a tertiary hospital of Uttar Pradesh concluded that those belonging to the general caste category were more likely to save money for birth related emergencies and that unfavourable pregnancy outcomes like abortion were much higher among pregnant women with birth preparedness index (consisting of knowledge on danger signs, transportation services and financial assistance provided by the government, identification of skilled birth attendant and mode of transportation for delivery, saving money, availing ANC services in 1^st^ trimester by a skilled provider) less than 50% [18]. Although these studies emphasized the importance of birth preparedness for safe deliveries i.e. childbirth in a health facility, in the state of Uttar Pradesh, most of these studies have limited generalizability due to small sample size and inadequately representative sample. There is also limited evidence on the association of antenatal care related factors with birth preparedness in the state. This present study aims to fill this gap by assessing the levels of birth preparedness, its determinants and its association with institutional deliveries in 25 High Priority Districts (HPDs) of Uttar Pradesh. Findings from this study would help in understanding the broader prevalence of birth preparedness in the state and associated factors in order to help guide health policy in the state and contribute towards strengthening the evidence base on birth preparedness in the country.

## Methods

### Study setting

This study was conducted in 25 HPDs of Uttar Pradesh with a population of 69.6 million accounting for 35% of the state population [19]. These HPDs were identified as poor performing districts by the Government of India (GoI) through the ranking of districts within all states based on a composite index of health impact and outcome indicators in the areas of maternal health, child health and family planning from the Annual Health Survey 2010–11 data [20]. This also includes 6 additional districts geographically contiguous to the 19 identified HPDs having poor maternal and child health indicators. The 25 HPDs in Uttar Pradesh, thus identified, are spread across different regions and are the highest contributors to maternal and infant mortality in the state. The University of Manitoba and India Health Action Trust (an India-based non-profit organisation) [21] established the Uttar Pradesh Technical Support Unit (UPTSU) to provide techno-managerial support for improving maternal, neonatal and child health outcomes in these HPDs. Within the 25 HPDs, 100 Community Development (CD) blocks equivalent to sub-districts, from 294 CD blocks were selected as focused CD blocks for intensification and as learning labs. The UPTSU provides mentoring support to Front Line Workers (FLWs) at the community level and clinical staff at the facility level as well as health systems strengthening support to ensure adequate availability, utilization and quality of antenatal, intra-partum and postnatal care services for women and children. Frontline health workers include the Accredited Social Health Activists (ASHAs) who are the closest available health functionary for the community. An ASHA covers a population of approximately 1000 people in a village and the closest available health facility for a village i.e. a sub-centre has around 10 ASHAs in its catchment area wherein a sub-centre caters to a population of almost 11,000 on an average in the state of Uttar Pradesh [21]. An ASHA is responsible for generating demand and mobilization of pregnant women for health services. This includes counselling them on birth preparedness, safe delivery and antenatal and postnatal care practices.

### Study design and participants

Community Behaviour Tracking Survey (CBTS), a cross-sectional quantitative survey, was conducted between June–October 2018 in rural areas of 25 HPDs of Uttar Pradesh by the UPTSU. The CBTS was designed to provide estimates of indicators of Reproductive, Maternal, Newborn and Child Health (RMNCH) program at block and districts levels in the selected geography. Accordingly, four population subgroups were chosen for data collection to measure different indicators under RMNCH. The survey methodology of CBTS is detailed elsewhere [22]. The Primary Sampling Unit (PSU) of this survey was the working area of an ASHA that caters to approximately 1000 people. Simple random sampling was used to select 20 CD blocks each among 100 and 194 CD blocks in 25 HPDs. Using a simple random sampling approach, 2811 primary sampling units (PSUs) within the selected blocks were chosen for one of the four survey groups i.e. the survey group with the maximum required number of PSUs. The required number of PSUs for the remaining survey groups were randomly selected from the already selected 2811 PSUs. For this analysis, data from a total of 2646 PSUs selected in Group-1 has been used. This process is depicted in Additional file 1. In each selected PSU, all households were visited to identify women who had a pregnancy outcome including live birth, stillbirth and abortion within 59 days preceding the survey. This timeframe was chosen to obtain more recent information to avoid possible recall bias. Overall, 13,908 women were identified based on the above criteria and 12,041 of them were interviewed (86.6% response rate). The majority of women not interviewed were not available during interview or had not returned from the facility or mother’s place (92.3%) and 0.7% had died during or after delivery or abortion. Among the interviewed women, 9458 women who had a delivery 2 months prior to the survey excluding those who had an abortion, were included in the analysis.

Research investigators were recruited and trained to administer a structured questionnaire in the local language (Hindi) to collect data on household and respondents’ background characteristics, relevant information on antenatal care, delivery care and postnatal care. The research investigators first determined the PSU boundary. With a random start, they visited all households and gave each household a number. They used a screening questionnaire in each household to identify women who recently completed a pregnancy, followed by informed verbal consent, and then, if eligible, proceeded with the implementation of the survey questionnaire.

### Variables

The respondents were asked “During this pregnancy where did you plan to deliver: at home or at any health facility?” and the responses were recorded as “at health facility”, “at home” and “not planned”. Similarly, the respondents were also asked “did you or your family identify in advance a vehicle you would use to reach health facility for delivery or in case of emergency?” and the responses were recorded as “Yes” or “No”. Based on these questions, a categorical variable for birth preparedness was computed as 1) identified both health facility and transport, 2) identified only health facility, 3) identified only transport, and 4) not planned. Women who planned for home delivery were included in the category “not planned”. Women were considered to have birth preparedness for this analysis if they identified both health facility and transport to reach the health facility for childbirth. The primary outcome variable, institutional delivery, is dichotomous (Yes, No) and defined as delivery conducted in a health facility, including both private and public facilities. The study included relevant confounders based on existing literature to understand the effect of birth preparedness on institutional delivery [12–18]. This includes the number of visits for antenatal check-ups during pregnancy (0, 1, 2, 3, 4 or more), number of times the ASHA contacted the women during pregnancy (no contact, 1–2 times, 3 or more times), 1^st^ trimester ANC registration (Yes, No), identified hypertension during pregnancy (Yes, No), identified anaemia during pregnancy (Yes, No), 3^rd^ trimester antenatal check-up (Yes, No) as well as background characteristics including maternal age (< 20, 20–24, 25–29, 30+), years of education (no education/< 5 years, 5–10 years, 10+ years), parity (1, 2, 3, 4+), religion (Hindu, non-Hindu), wealth quintile (poorest, poorer, middle, richer, richest), caste (Scheduled Caste (SC)/ Scheduled Tribe (ST), Other Backward Class (OBC), Others). Caste is a social stratification system based on heredity that has existed historically in India. A caste can be equivalent to social class in India where the ‘backward class’ in Article 16 (4) of the constitution of India refers to OBC, SC and ST based on social backwardness which leads to economic and educational backwardness [23, 24]. The caste category “Others” has been used to denote castes whose members are economically and socially advantaged as compared to the socially disadvantaged caste category members (SC/ST, OBC). SC and ST caste categories for any state or union territory may be defined by the government of India through a public notification and the OBC list is notified by the National Commission for Backward Classes. Hence, while caste may be synonymous with socio-economic status, the wealth quintile has been considered as an appropriate methodology for quantifying it. Wealth quintile was computed based on the composite score derived from a set of household indicators such as access to drinking water (tap water, hand pump/protected well, others), access to toilet facility (flush, pit, no toilet), type of dwelling (pucca, semi-pucca, kaccha) and ownership of household assets (electricity, black & white television, colour television, mobile, land telephone, refrigerator, air conditioner, bicycle, motorcycle, car, water pump, tractor) using Principal Component Analysis (PCA) [25]. Participants were then categorized into five wealth quintiles: the first 20^th^ percentile group, representing the relatively poorest quintile of the participants and 5^th^ quintile, representing the relatively richest participants.

### Data analysis

Descriptive analysis was conducted to describe the background characteristics of the pregnant women and estimate the proportion of pregnant women with birth preparedness. Bivariate analysis and multivariate logistic regressions were used to test the association of background characteristics and antenatal care related factors with birth preparedness as well as that of birth preparedness with institutional deliveries. Multivariate results are presented in the form of adjusted odds ratios (aOR) with 95% confidence interval (95% CI). All analyses were done using STATA version 15.1 [26].

## Results

### Profile of respondents

Among the 9458 respondents, majority were Hindus (81.9%), 57.6% belonged to the Other Backward Classes (OBC) category and 54.6% had less than 5 years of education. While only 45% of pregnant women were registered for antenatal care in their 1^st^ trimester and 31.5% received ANC check-ups more than four times, the majority received an ANC check-up in their 3^rd^ trimester (71.4%). Overall, 61.8% of women had a birth preparedness plan and 79.1% of women delivered in a health facility with the majority (60.8%) delivering in public facilities (Additional file 2).

### Birth preparedness and its determinants

Table 1 shows that a relatively higher proportion of pregnant women who identified as Hindu (62.9%) and those belonging to “others” caste category (67.9%) had a birth preparedness plan compared to their respective counterparts. Women with 10 or more years of education, women who gave birth four or more times (57%) and those belonging to the wealthiest quintile (68.7%) were more prepared for birth as compared to non-literate (55.5%), first parity (63.3%) women and those belonging to the poorest wealth quintile (56.6%) families. Registration in the first trimester for antenatal care was also significantly associated with birth preparedness where 66.3% of pregnant women were registered in their 1^st^ trimester. Women who received more than four ANC check-ups (70.8%) had higher birth preparedness as compared to those who did not receive any ANC check-up (40.1%). ASHA contact during the ANC period was also a significant predictor of birth preparedness where 70.6% of pregnant women who were contacted by ASHAs more than 3 times were prepared for birth as compared to only 55.5% of women with no ASHA contact.

**Table 1:** Association between individual, household and antenatal care related characteristics with birth preparedness among recently delivered women (*N* = 9458)

Birth Preparedness Plan (1- Identified facility & transport both, Else-0)							
Characteristic	Identified facility and transportation (%)	Identified only facility (%)	Identified only transport (%)	Not planned (%)	N	***P***-value	Adjusted Odds Ratio[95% CI]
Age						0.000	
30 and above (Ref.)	54.8	11.2	16.9	17.1	675		
< 20	62.6	10.4	12.6	14.4	4233		0.83*[0.7–0.99]
20–24	64.8	10.6	10.1	14.5	3030		1.26**[1.13–1.42]
25–29	56.4	11.9	11.3	20.4	1520		1.06 [0.9–1.24]
Religion						0.001	
Hindu (Ref.)	62.9	10.4	11.8	15.0	7816		
Non Hindu	57.1	12.5	12.6	17.9	1642		0.83*[0.74–0.94]
Caste						0.000	
SC/ST (Ref.)	61.4	11.6	11.6	15.4	2623		
OBC	60.6	10.6	12.2	16.6	5528		0.92 [0.83–1.02]
Others	67.9	9.5	11.4	11.1	1307		1.24*[1.06–1.45]
Years of education						0.000	
No-education / < 5 years (Ref.)	55.5	11.1	13.2	20.3	5387		
5–10 years	67.0	10.3	11.4	11.3	2387		1.42**[1.28–1.58]
10+ years	72.7	10.4	9.0	7.8	1684		1.68**[1.46–1.92]
Parity						0.000	
1 (Ref.)	63.3	10.6	13.3	12.8	2984		
2	64.6	10.2	10.7	14.6	2382		1.11 [0.98–1.25]
3	61.5	10.4	12.5	15.6	1764		1.05 [0.91–1.22]
4+	57.0	11.8	11.0	20.3	2328		1.04 [0.88–1.22]
Poverty status						0.000	
Poorest (Ref.)	56.6	10.8	10.9	21.6	1969		
Poorer	59.3	11.0	13.4	16.3	1856		0.96 [0.84–1.1]
Middle	60.3	11.0	12.7	16.1	1882		0.95 [0.82–1.08]
Richer	64.2	11.2	11.3	13.4	1880		0.98 [0.85–1.13]
Richest	68.7	9.7	11.3	10.3	1871		1.02 [0.87–1.19]
1^st^ trimester registration						0.000	
No (Ref.)	66.3	9.8	11.5	12.4	4227		
Yes	58.1	11.5	12.3	18.1	5231		1.14*[1.04–1.25]
Number of ANC check-ups						0.000	
4+ (Ref.)	40.1	9.8	13.9	36.3	1157		
0	53.3	9.9	15.0	21.8	1383		0.41**[0.34–0.5]
1	62.3	10.3	12.4	15.0	2154		0.64**[0.54–0.75]
2	65.7	11.6	11.3	11.5	1898		0.84*[0.74–0.96]
3	70.8	11.2	9.9	8.1	2866		0.9 [0.8–1.03]
3^rd^ trimester ANC check-up						0.000	
No (Ref.)	66.1	10.9	11.3	11.7	6623		
Yes	51.0	10.3	13.5	25.2	2835		1.05 [0.92–1.19]
Number of times ASHA contacted during ANC						0.000	
No contact (Ref.)	55.5	11.2	13.1	20.3	4034		
1–2 contact	57.8	10.0	13.6	18.5	1917		1.07 [0.95–1.19]
3+	70.6	10.7	9.8	8.9	3507		1.61**[1.46–1.79]
Identified with Hypertension						0.029	
No (Ref.)	65.3	12.2	11.0	11.5	474		
Yes	61.6	10.7	12.0	15.8	8984		0.88 [0.72–1.08]
Identified with Anaemia						0.000	
No (Ref.)	67.0	10.0	13.2	9.8	1970		
Yes	60.4	10.9	11.6	17.0	7488		1.07 [0.96–1.2]

*Note*: The above model is adjusted for age, religion, caste, years of education, parity, poverty status, 1^st^ trimester ANC registration, number of ANC check-ups, 3^rd^ trimester ANC check-up, number of times ASHA contacted during pregnancy, identified with anemia and identified with hypertension

*CI* Confidence Interval, *Ref* Reference Category.

*p < 0.05, **p < 0.001

Findings reveal that, as compared to women belonging to SC/ST caste category, women from others caste category were 24% more likely to have a birth preparedness plan (aOR = 1.24, CI 1.06–1.45). Women in the age category of 20–24 years (aOR = 1.26, CI 1.13–1.42) compared to those aged 30 and above were significantly more likely to have a birth preparedness plan. Women with more than 10 years of education (aOR = 1.68, CI 1.46–1.92) and those who registered their pregnancy in first trimester (aOR = 1.14, CI 1.04–1.25) were significantly more likely to have a birth preparedness plan compared to women with less than 5 years of education and those who did not register their pregnancy in first trimester respectively. Women with three or more times contacts with ASHAs (aOR = 1.61, CI 1.46–1.79) were also found to have significantly higher odds of birth preparedness in comparison with women who had no contact with ASHA during ANC. Further, women who received no ANC services (aOR = 0.41, CI 0.34–0.5) were significantly less likely to have a birth preparedness plan as compared to women who received ANC services 4 or more times.

### Association of birth preparedness with institutional delivery

To examine the association of birth preparedness and other factors with institutional delivery, bivariate and multivariate analysis was conducted (Table 2). The results indicated a significant association between birth preparedness and institutional delivery where 89.1% of pregnant women who had identified both facility and transportation for childbirth, delivered in a health facility. Multivariate analysis of institutional delivery by birth preparedness depicted that after controlling for other variables included in the model, women who had a birth preparedness plan were seven times more likely to have an institutional delivery (aOR = 7.00, CI 6.07–8.08) and those who identified only a facility for birth had five times higher odds of undergoing an institutional delivery (aOR = 5.38, CI 4.36–6.65) as compared to those who did not prepare.

**Table 2:** Institutional delivery by birth preparedness and other factors

Characteristic	%	N	Unadjusted OR	95% CI	Adjusted OR		95% CI	
Birth Preparedness								
Not planned (Ref.)	46.8	1617	1.00			1.00		
Identified facility and transportation	89.1	5710	9.25**	8.12	10.54	7.00**	6.07	8.08
Identification of only facility	84.8	933	6.35**	5.21	7.75	5.38**	4.36	6.65
Identification of only transport	64.1	1198	2.03**	1.74	2.38	1.59**	1.34	1.89

Note: The above model is adjusted for religion, caste, years of education, parity, poverty status, 1^st^ trimester ANC registration, number of ANC check-ups, 3^rd^ trimester ANC check-up, number of times ASHA contacted during pregnancy, and women with anemia

*CI* Confidence Interval, *Ref* Reference Category

**p < 0.001, *p **< 0.05**

## Discussion

This study assesses the determinants of birth preparedness among pregnant women and its effect on institutional delivery in the high priority districts of Uttar Pradesh, India. The results of this study suggest that birth preparedness in Uttar Pradesh varies significantly based on socioeconomic background as well as antenatal care service-related factors. About two-thirds of pregnant women (61.8%) had identified both a facility and transport for childbirth and 79.1% underwent institutional delivery. Pregnant women’s religion, caste, education level and parity were found to have a strong association with birth preparedness. Among the antenatal care related factors, early registration of pregnant women for antenatal care and increased ASHA contacts during the antenatal period improved birth preparedness significantly. Pregnant women with a birth preparedness plan (those who identified both facility and transport for childbirth) were found to be seven times more likely to undergo delivery in health facility.

The study findings also suggest that pregnant women belonging to OBC and SC/ST caste category have lower birth preparedness levels and are consequently less likely to opt for delivery in a health facility. This is corroborated by a previous study in the state of Uttar Pradesh that demonstrated that women belonging to SC/ST and OBC castes are less likely to undergo an institutional delivery as compared to Other caste women, despite a lack of association between caste and ASHA visits [27]. A qualitative study by Blanchard et al. suggests that families belonging to lower socio-economic strata are usually geographically clustered in distant hamlets or villages with less infrastructure leading them to face greater delays and financial barriers in accessing private facilities nearby or higher-level public services [28]. Furthermore, higher education increases the odds of a pregnant woman being prepared for birth, consistent with other data from India [29–31]. Since, women belonging to marginalized groups contribute to a large proportion of less or uneducated women, they are less likely to be prepared for childbirth, consequently leading to a lower likelihood of institutional delivery among these women. Hence, there is a need to prioritize less educated or illiterate women, particularly those belonging to marginalized castes, for counselling on birth preparedness and increase use of pictorial materials, video and audio-based messages for better understanding and usability. Experience sharing by women with a previous institutional delivery during mothers’ group meetings could also be helpful in building confidence on institutional delivery among other women.

The study also suggests that higher parity women (4+ parity) have lower odds of birth preparedness and are less likely to undergo institutional deliveries, as noted in other similar studies [31]. Lesser risk perception and poor previous experience of delivery [32] could be contributing factors towards fewer institutional deliveries among higher parity women. Improved communication by health care providers and frontline workers on the importance of delivering in a health facility and increased risk of complications with increased parity along with improved quality and experience of care in facilities could be beneficial in increasing coverage of institutional delivery among higher parity women [33, 34].

Beyond the individual level factors associated with birth preparedness, our data supports the need to increase antenatal care visits, early registration and higher ASHA contacts through addressing health system level constraints and ensuring access and availability of quality services. As per WHO recommendations [35], the first contact between a pregnant woman and health care provider should be in the first trimester of pregnancy as early registration facilitates increasing the coverage of antenatal care visits among pregnant women. Early registration also helps in timely diagnosis, prevention and treatment of pregnancy related morbidities [36]. The results of this study highlight the need for improving first trimester registration among pregnant women, as this can increase the probability of women being prepared for childbirth. This can be achieved through improving the availability of pregnancy test kits with front line workers such as ASHAs for early confirmation of pregnancy. There is also a need to improve enumeration, counselling and regular follow-up of the last menstrual period of women of reproductive age for early identification of pregnancy. Increasing awareness on entitlements and government schemes like Pradhan Mantri Matru Vandana Yojana (PMMVY) that provide structured incentives to pregnant women for early registration could also be helpful in improving the rates of early registration among pregnant women [37].

As seen in previous studies on birth preparedness conducted in Africa [12, 13] and elsewhere in India [30], the number of ANC check-ups are significantly associated with birth preparedness amongst pregnant women in this study. Antenatal care sessions, whether held at a facility or as part of monthly village level outreach activities, provide an opportunity to reinforce messages on birth preparedness and hence, it is important for health programs to focus on effective counselling by healthcare providers as well as FLWs during these sessions. Since currently, less than one third of pregnant women have 4 or more ANC visits, ASHAs’ efforts in motivating women for a minimum of four ANC check-ups can be critical. It is important to note the role that FLWs play in generating awareness, promoting birth preparedness and counselling for institutional deliveries during the antenatal period. Further, the finding that increased frequency of ASHA contacts positively affects the odds of birth preparedness strengthens the argument for improved engagement of FLWs for counselling. Hence, there is a need to invest in building knowledge and counselling skills of ASHAs and other FLWs as well as effectively utilizing antenatal care service delivery platforms for improved counselling on birth preparedness.

Antenatal care in the third trimester of pregnancy is also a key determinant of institutional delivery. The birth preparedness plan is usually reviewed by healthcare providers during the third trimester of pregnancy and may be revised based on the status of the pregnant woman. Hence, contact with pregnant women during the third trimester provides an important opportunity to emphasize on messaging related to birth preparedness and safe deliveries. While pregnant women identify the mode of transport for delivery during this time, it is also important to work towards ensuring timely and easy availability of ambulance services through system level interventions.

This study does have some important limitations, including the possibility of a recall bias as the study is retrospective in nature. We tried to minimize this by keeping the reference period short (60 days preceding the survey). The cross-sectional design of the study does not allow establishing causality since the study lacks temporal information. Moreover, the study did not collect data on other aspects of birth preparedness like saving money, identification of blood donor and other factors that could affect the comprehensiveness of birth preparedness.

## Conclusion

The findings from this study highlight the importance of birth preparedness for improving institutional deliveries in the state of Uttar Pradesh for improved maternal and newborn outcomes. The study findings further highlight the need for a greater emphasis on marginalized populations (including those from backward castes and with less education) at the policy and implementation levels to improve the overall institutional delivery rates in the state. Government, as well as health partners and funding agencies investing in the state, need to focus on enhancing equity in health service delivery. Efforts need to be made towards improving early registration of pregnancies in order to increase FLW contacts and ANC coverage, subsequently leading to improved birth preparedness. Antenatal care service delivery platforms and home visits made by ASHAs to the pregnant women should be effectively utilized to ensure all pregnant women receive counselling on birth preparedness, particularly in the third trimester. Furthermore, the study highlights the important role that FLWs play in ensuring birth preparedness and institutional delivery. Hence, mechanisms for improving knowledge and counselling skills of FLWs on birth preparedness need to be incorporated into the existing FLW capacity building processes to improve birth preparedness and subsequently institutional delivery rates.

## Supplementary Information

**Additional file 1.** Framework of sample selection in the survey. This file depicts the methodology of the survey in detail through a flow diagram.

**Additional file 2. **Socio-economic background and obstetric characteristics of the respondents (*N* = 9458). This file represents the profile of the respondents of the survey that includes their socio-economic background, antenatal care related factors, overall birth preparedness and institutional delivery rates.

## Data Availability

The datasets used and/or analysed during the current study are available from the corresponding authors on reasonable request.

## Abbreviations

ANC: Ante Natal Care
aOR: Adjusted Odds Ratio
ASHA: Accredited Social Health Activist
BMGF: Bill and Melinda Gates Foundation
CI: Confidence Interval
FLW: Frontline Worker
GoUP: Government of Uttar Pradesh
HPD: High Priority Districts
JHPIEGO: John Hopkins Program for International Education in Gynaecology and Obstetrics
MMR: Maternal Mortality Ratio
NMR: Neonatal Mortality Rate
OBC: Other Backward Caste
RMNCH: Reproductive, Maternal, Newborn and Child Health
SC/ST: Scheduled Caste/Scheduled Tribe
UoM: University of Manitoba
UPTSU: Uttar Pradesh Technical Support Unit
WHO: World Health Organization
