# Supplementary material for: Association of identification of facility and transportation for childbirth with institutional delivery in high priority districts of Uttar Pradesh, India
Source: BMC Pregnancy Childbirth. 2021 Oct 27;21:724. doi: 10.1186/s12884-021-04187-5 (PMC8549204; doi:10.1186/s12884-021-04187-5)
Supplement: Supplementary file 1 — Additional file 1. Framework of sample selection in the survey. This file depicts the methodology of the survey in detail through a flow diagram. [file 12884_2021_4187_MOESM1_ESM.docx]

25 High Priority Districts, UP

(294 CD Blocks)

100 CD Blocks

194 CD Blocks

20 CD Blocks selected randomly

20 CD Blocks selected randomly

13908 women who had a pregnancy outcome (still birth, live birth, abortion) identified

PSUs for the remaining survey groups were randomly selected from the already selected PSUs.

Total 2646 PSUs in group-1 were considered for this analysis (1312 PSUs selected out of 1362 selected PSUs and 1334 PSUs selected out of 1449 selected PSUs)

12041 women were interviewed

1362 PSUs (ASHA areas) were selected randomly for one of four survey groups with maximum required number of PSUs from a complete listing of ASHA areas in the block

1449 PSUs (ASHA areas) were selected randomly for one of four survey groups with maximum required number of PSUs from a complete listing of ASHA areas in the block

1723 women were not available for interview, 13 had died and the rest denied the interview or had other reasons

2583 women had an abortion

9458 women considered for this analysis
